# Supplementary material for: Development of Optimized Vitrification Procedures Using Closed Carrier System to Improve the Survival and Developmental Competence of Vitrified Mouse Oocytes
Source: Cells. 2021 Jul 2;10(7):1670. doi: 10.3390/cells10071670 (PMC8304188; doi:10.3390/cells10071670)
Supplement: Supplementary file 1 [file cells-10-01670-s001.zip › cells-1266830-supplementary.pdf]

cells

Supplementary Material

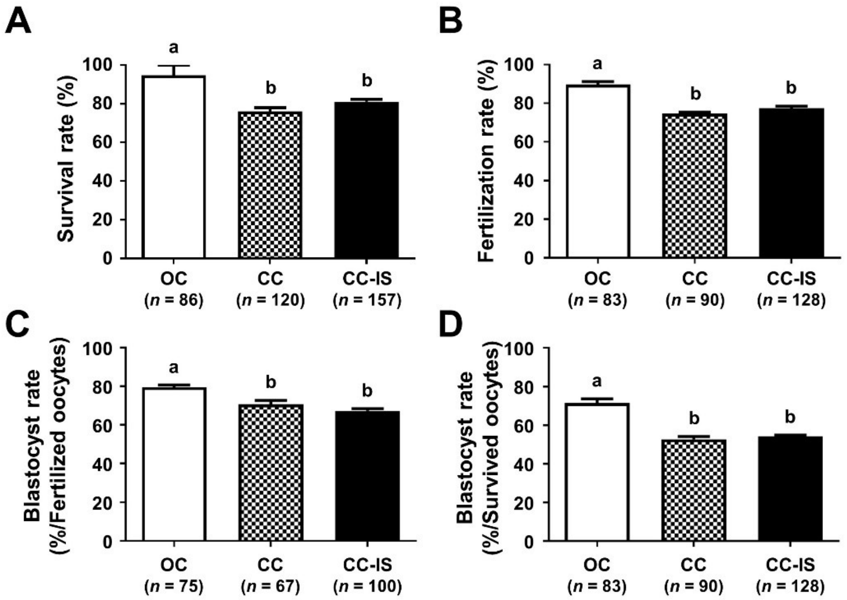

Figure S1. Comparison of the survival and development rates of vitrified/warmed (VW) oocytes with increased sucrose concentration. Comparison of the survival (**A**), fertilization (**B**), and blastocyst formation rates (**C**–**D**) of VW oocytes. Blastocyst formation rates [the number of blastocysts/the number of fertilized oocytes (**C**) and /the number of survived oocytes (**D**)]. Different superscript letters (a,b) indicate $p < 0.05$. The number of experiments = 4 (28–40 oocytes per each group in each experiment). OC, open carrier system; CC, closed carrier system with standard sucrose concentration (0.5 M); CC-IS, increased sucrose concentration (1.0 M).

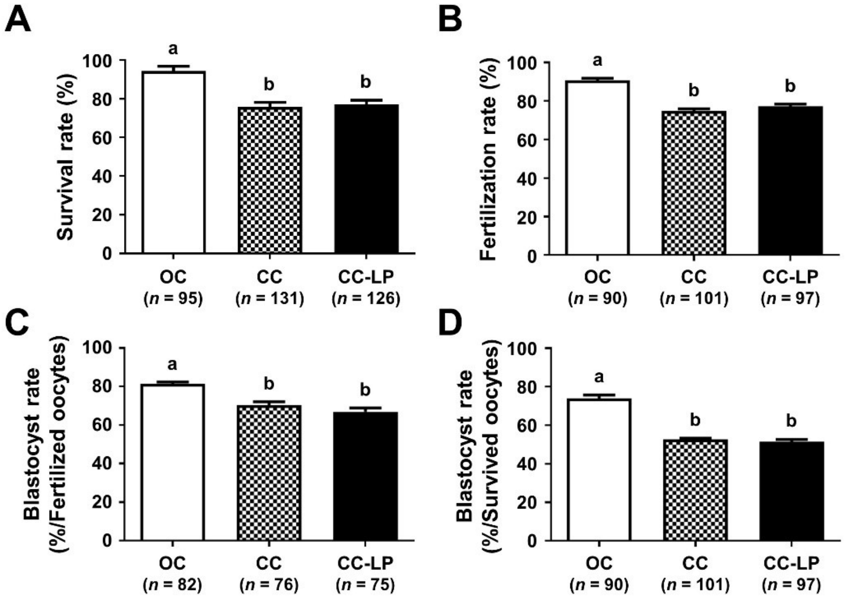

Figure S2. Comparison of the survival and development rates of vitrified/warmed (VW) oocytes with increased viscosity by L-proline supplementation. Comparison of the survival (**A**), fertilization (**B**), and blastocyst formation rates (**C**–**D**) of VW oocytes. Blastocyst formation rates [the number of blastocysts/the number of fertilized oocytes (**C**) and /the number of survived oocytes (**D**)]. Different superscript letters (a,b) indicate $p < 0.05$. The number of experiments = 4 (22–31 oocytes per each group in each experiment). OC, open carrier system; CC, closed carrier system without L-proline; CC-LP, L-proline supplementation as a cryoprotective agent.
